# Characterization of a Linezolid- and Vancomycin-Resistant *Streptococcus suis* Isolate That Harbors *optrA* and *vanG* Operons

**DOI:** 10.3389/fmicb.2019.02026

**Published:** 2019-09-10

**Authors:** Fanshu Du, Xi Lv, Duan Duan, Liping Wang, Jinhu Huang

**Affiliations:** MOE Joint International Research Laboratory of Animal Health and Food Safety, College of Veterinary Medicine, Nanjing Agricultural University, Nanjing, China

**Keywords:** *Streptococcus suis*, vancomycin resistance, *vanG*, linezolid resistance, *optrA*, mobile genetic elements, pathogenicity

## Abstract

Linezolid and vancomycin are among the last-resort antimicrobial agents in the treatment of multidrug-resistant Gram-positive bacterial infections. Linezolid- and vancomycin-resistant (LVR) Gram-positive bacteria may pose severe threats to public health. In this study, three *optrA-* and *vanG*-positive *Streptococcus suis* strains were isolated from two farms of different cities. There were only 1 and 343 single-nucleotide polymorphisms in coding region (cSNPs) of HCB4 and YSJ7 to YSJ17, respectively. Mobilome analysis revealed the presence of *vanG*, *erm*(B), *tet*(O/W/32/O), and *aadE*-*apt*-*sat4*-*aphA3* cluster on an integrative and conjugative element, ICE*Ssu*YSJ17, and *erm*(B), *aphA3*, *aac(6′)-aph(2″)*, *cat*pC_194_, and *optrA* on a prophage, ΦSsuYSJ17-3. ICE*Ssu*YSJ17 exhibited a mosaic structure and belongs to a highly prevalent and transferable ICE*Sa*2603 family of *Streptococcus* species. ΦSsuYSJ17-3 shared conserved backbone to a transferable prophage Φm46.1. A novel composite transposon, IS*1216E*-*araC*-*optrA*-*hp*-*cat*pC_194_-IS*1216E*, which can be circulated as translocatable unit (TU) by IS*1216E*, was integrated on ΦSsuYSJ17-3. Vancomycin resistance phenotype and *vanG* transcription assays revealed that the *vanG* operon was inducible. The LVR strain YSJ17 exhibited moderate virulence in a zebrafish infection model. To our knowledge, this is the first report of LVR isolate, which is mediated by acquired resistance genes *optrA* and *vanG* operons in Gram-positive bacteria. Since *S. suis* has been recognized as an antimicrobial resistance reservoir in the spread of resistance genes to major streptococcal pathogens, the potential risks of disseminating of *optrA* and *vanG* from *S. suis* to other *Streptococcus* spp. are worrisome and routine surveillance should be strengthened.

## Introduction

*Streptococcus suis* is one of the most important bacterial causes of meningitis and contributes substantially to antimicrobial use in swine industry worldwide (Goyette-Desjardins et al., 2014). As a normal colonizer of the respiratory tract in pigs, *S. suis* can cause serious invasive infections in both pigs and humans, which posed a major public health challenge in Southeast Asia, including China (Tang et al., 2006; Yu et al., 2006). *S. suis* is thought to be an important antimicrobial resistance (AMR) reservoir contributing to the spread of AMR genes to major streptococcal pathogens (Palmieri et al., 2011; Huang et al., 2016b).

Avoparcin, a vancomycin analog, was widely used in food animals as a feed additive during 1940–1990s. The misuse of avoparcin had been reported to be associated with the occurrence of vancomycin-resistant enterococcus (VRE) in most parts of Europe (Bager et al., 1997; Hao et al., 2016). For this reason, avoparcin has been banned in food-producing animals in Denmark, 1995, and subsequently in China, 2002. However, vancomycin resistance still persists in pig farms even 20 years after the ban of avoparcin (Bortolaia et al., 2015; Birkegard et al., 2019). Two groups of *van* resistance operons have been identified according to the key ligase genes that encode either D-Ala-D-Lac ligase (*vanA*, *vanB*, *vanD*, and *vanM*) or D-Ala-D-Ser ligase (*vanC*, *vanE*, *vanG*, *vanL*, and *vanN*) (Lebreton et al., 2011; Binda et al., 2014). The *vanG* operon confers low-level vancomycin resistance and has been detected in many species, including *Enterococcus faecalis*, *Enterococcus faecium*, *Clostridium difficile*, *Clostridium argentinense*, *Ruminococcus*, and *Streptococcus agalactiae* (Depardieu et al., 2003; Domingo et al., 2007; Ammam et al., 2012; Srinivasan et al., 2014; Berthet et al., 2015; Sassi et al., 2018). Recently, we reported the *vanG*-type vancomycin resistance in zoonotic pathogen *S. suis* (Huang et al., 2018).

Oxazolidinones, including linezolid and tedizolid, are recognized as last-resort antimicrobial agents for the control of clinical infections caused by multidrug-resistant Gram-positive pathogens. Linezolid resistance in these bacteria is traditionally associated with mutations in domain V of *23S rRNA* gene and ribosomal proteins L3 and L4 (Mendes et al., 2014). However, transferable resistance genes, *cfr*, *cfr*(B), and *optrA*, have been identified in enterococci worldwide, including Jiangsu, China (Long et al., 2006; Deshpande et al., 2015; Wang et al., 2015; Antonelli et al., 2018; Bender et al., 2018; Zhou et al., 2019), which also confer resistance to phenicols and other ribosomal-targeted antibiotics. More recently, a third oxazolidinone resistance gene, *poxtA*, was identified from MRSA and enterococci (Antonelli et al., 2018; Brenciani et al., 2019; Huang et al., 2019b; Lei et al., 2019). Currently, only *cfr* and *optrA* have been reported in streptococci, only in *S. suis* of animal origin (Wang et al., 2013; Huang et al., 2017), thus suggesting that *cfr* and *optrA* have occurred in animal setting under selection, as phenicols and other ribosomal-targeted antibiotics are broadly used in veterinary medicine (Shen et al., 2013; Hao et al., 2016).

Linezolid- and vancomycin-resistant (LVR) enterococci in hospitals have been recently described (O’driscoll et al., 2015; Krull et al., 2016; Bender et al., 2018). However, researches on other Gram-positive bacteria and in other settings have not been documented. In this study, to the best of our knowledge, we present the first LVR *S. suis* isolate of pig origin, which was mediated by *optrA* and *vanG* operons. The genetic basis of *optrA* and *vanG* operons was characterized by whole-genome sequencing (WGS) and the virulence was evaluated using a zebrafish infection model.

## Materials and Methods

### Sample Processing and Bacteria Identification

One hundred and eighty-nine *S. suis* clinical isolates used in this study were collected from a total of 658 pig samples, including pharyngeal swabs from 600 asymptomatic pigs and tissues (heart, liver, spleen, lung, tonsil, and joint fluid each pig) from 58 diseased pigs, Jiangsu Province, China, 2016–2017. This study aims to survey the current status of oxazolidinone resistance in *S. suis* from pigs. The swabs and tissues were collected by farm veterinary and delivered to the laboratory within 24 h. The samples were plated on the Todd–Hewitt agar supplemented with 5% fetal calf serum in the presence of nalidixic acid (15 mg/L) and Polymyxin B (10 mg/L). Five colonies each sample were selected and cultured in Todd–Hewitt broth with 5% fetal calf serum followed by PCR identification by using *S. suis*-specific primers targeting *gdh* and *recN* genes (Ishida et al., 2014). Duplicate isolates from samples of the same pig were excluded.

### Antimicrobial Susceptibility Assays and LVR Mechanisms

The MICs of vancomycin (VAN), linezolid (LZD), and florfenicol (FFC) as well as penicillin (PEN), enrofloxacin (ENR), gentamicin (GEN), streptomycin (SPT), kanamycin (KAN), tetracycline (TET), erythromycin (ERY), tilmicosin (TIL), and lincomycin (LIN) to *S. suis* isolates were tested and evaluated by the broth microdilution method according to the Clinical and Laboratory Standards Institute guidelines (VET08-ED4 and M100-ED28). The presence of LVR resistance genes *vanG*, *cfr*, *cfr*(B), *cfr*(C), *optrA*, and *poxtA* was detected by PCR using primers previously described (Supplementary Table S1).

### Genome Sequencing and Analysis

Genomic DNA was prepared from overnight cultures of the LVR isolates using the E.Z.N.A.^®^, Bacteria DNA kit (Omega Bio-Tek, Nanjing, China). Purified genomic DNA was subjected for WGS on the Illumina Hiseq2500 platform (Novogene, Beijing, China). Draft genome was assembled with SOAP *de novo* version 2.04 by default parameters (Li et al., 2008). For complete genome sequencing, the genomic DNA was further sequenced using the PacBio RSII System (Biozeron, Shanghai, China). The sequences were annotated using the NCBI Prokaryotic Genome Annotation Pipeline.

Average nucleotide identity (ANI) analysis was performed using the pyani software^1^ using whole genome sequences. The single-nucleotide polymorphisms in coding region (cSNPs) were determined by global alignment and local alignment between sample sequence and the reference genome. The maximum-likelihood (ML) methods were performed for the genome-wide phylogenetic analysis using PhyML 3.0 (Guindon et al., 2010). Nucleotide substitution model selection was estimated with jModelTest 2.1.10 (Darriba et al., 2012) and Smart Model Selection in PhyML 3.0. The model GTR + G was selected for ML analyses with 1,000 bootstrap replicates to calculate the bootstrap values of the topology.

Additional acquired resistance genes were identified in the genomes using ResFinder 3.1 (Zankari et al., 2012). Integrative and conjugative elements (ICEs) and prophages in NCL1 strain YSJ17 were identified by comparison with other NCL1 strains from this study and GenBank, and representative genomes of *S. suis* serotype 2, 9, and 24. Chromosomal mutations involved in β-lactam resistance and substitutions responsible for fluoroquinolone resistance were identified using BLASTn analysis.

### Transferability Assays

Conjugative transfer assays were examined by filter mating experiment as described previously (Huang et al., 2016a). In mating experiments, donor and recipient strains were mixed at a ratio of 1:10 on a nitrocellulose membrane. Selection of transconjugants was performed on Todd–Hewitt agar containing rifampin (25 mg/L), fusidic acid (50 mg/L), and florfenicol (10 mg/L) or vancomycin (1 mg/L). Strain *S. suis* YSJ17 served as donor and *S. suis* P1/7RF (also known as BAA-853RF) was used as recipient (Huang et al., 2016b). Control, donor, and recipient strains were plated on selective medium independently. In addition, an overlap and inverse PCR method was introduced to detect the circular intermediate form of the novel *optrA*- and *cat*pC_194_-carrying IS*1216* composite transposons and the *vanG*-carrying ARGI2 using primer pairs (Supplementary Table S1).

### Inducible Vancomycin Resistance Assays

Inducible vancomycin resistance phenotype assay was performed as previously described (Srinivasan et al., 2014; Huang et al., 2018). In brief, strains were pre-incubated with 1/10 × MIC vancomycin for 1 h in THB supplemented with 0.2% yeast extract (THY). Bacterial cultures were then diluted to 0.05 of OD_600_ in THY with 1/2 × MIC vancomycin. The growth curve (*A*_600_) was measured every hour over a 13-h period. Inducible transcription of the *vanG* gene was investigated using RT-PCR with RNA templates extracted from bacterial cultures in the absence or incubation with 1/10 × MIC or 1/2 × MIC of vancomycin. RNA isolation and PCR amplification were carried out as described previously (Huang et al., 2018).

### Ethics Statement and Zebrafish Infection Model

The zebrafish infection experimental protocols were handled according to the guidelines of Experimental Animal Management Measures of Jiangsu Province and were approved by the Laboratory Animal Monitoring Committee of Jiangsu Province, China [Permit number: SYXK (Su) 2017-0007]. The zebrafish infection experiments were carried out as previously reported (Wu et al., 2010). Five groups of 15 zebrafish each were injected with 20 μL of PBS or bacterial suspensions containing a series of 10-fold serial dilutions (10^5^–10^8^ cfu), respectively, incubated in plastic containers for 72 h at 28°C, and the mortality was monitored from three parallel experiments. The LD_50_ values at 72 h were calculated by the Reed and Muench (1938) method.

## Results

### Isolation of *vanG*- and *optrA*-Positive *S. sui*s

Since the first report of *vanG* operon in a *S. suis* serotype 24 isolate (Huang et al., 2018), we began the experiment by looking for the prevalence of *vanG* operon in *S. suis* using previously reported primers (Supplementary Table S1). Among 189 *S. suis* isolates collected from Jiangsu, China, 3 strains of different pig origin were positive for *vanG*, but exhibited variable phenotype to vancomycin. Strain YSJ17 (Farm YS) was vancomycin non-susceptible (MIC 2 mg/L), while YSJ7 (Farm YS) and HCB4 (Farm HC) were vancomycin susceptible (MIC 0.5 mg/L). On the other hand, 68 (35.98%) of the 189 isolates were *optrA-*positive (Table 1), with linezolid MIC values ranging from 0.25 to 16 mg/L. Among them, one isolate was also positive for *cfr* (Huang et al., 2019a) and none of the isolates was positive for *cfr*(B), *cfr*(C), or *poxtA*.

**TABLE 1:** Results of the screening of 189 *Streptococcus suis* of pig origin in Jiangsu, China, for the presence of the *vanG*, *cfr*, and *optrA* genes.

**City**	**Total number of isolates**	**Number (percentages) of *vanG*-positive isolates**	**Number (percentages) of *cfr*-positive isolates**	**Number (percentages) of *optrA*-positive isolates**
Nantong	6			3 (50.00%)
Huai’an	31	2 (6.45%)^a^	1 (3.23%)^a^	9 (29.03%)
Suqian	67	1 (1.49%)^a^		13 (19.40%)
Yancheng	85			43 (50.58%)
–	189	3 (1.59%)	1 (0.53%)	68 (35.98%)

*^*a*^These isolates also carried *optrA* gene.*

It is noteworthy that all three *vanG*-carrying isolates were linezolid non-susceptible and carried the *optrA* gene (Table 2). In addition, they exhibited resistance or elevated MIC values to penicillin, enrofloxacin, gentamicin, streptomycin, kanamycin, tetracycline, erythromycin, tilmicosin, lincomycin, and florfenicol (Table 2). Since, to our knowledge, no *vanG*- and *optrA*-positive bacteria have been identified so far, we further analyzed these *S. suis* strains by WGS.

**TABLE 2:** Principal features of the *S. suis* isolates carrying *vanG* and *optrA.*

**Strain**	**Isolation**	**Source**	**Serotype, ST**	**MICs (mg/L)^a^**											
				**VAN**	**LZD**	**FFC**	**PEN**	**ENR**	**GEN**	**SPT**	**KAN**	**TET**	**ERY**	**TIL**	**LIN**
YSJ17	Huai’an, Jiangsu, 20 October 2016	Nasal swab, Farm YS	NCL1, ST-1071	2	4	32	4	32	>256	>256	128	256	>256	>256	256
YSJ7	Huai’an, Jiangsu, 20 October 2016	Nasal swab, Farm YS	NCL1, ST-1071	0.5	4	8	4	16	>256	>256	128	64	>256	>256	>256
HCB4	Suqian, Jiangsu, 21 November 2016	Tonsil, Farm HC	NCL1, ST-1071	0.5	2	4	0.5	16	256	>256	64	32	>256	>256	>256

*^*a*^The MIC breakpoint accords to the guidelines for *S. suis* or other *Streptococcus* spp. of the CLSI (VET08-ED4 or M100-ED28). VAN, vancomycin; LZD, linezolid; FFC, florfenicol; PEN, penicillin; ENR, enrofloxacin; SPT, streptomycin; GEN, gentamicin; KAN, kanamycin; TET, tetracycline; ERY, erythromycin; TIL, tilmicosin; LIN, lincomycin.*

### Genomic and Phylogenetic Analyses

**FIGURE 1:**
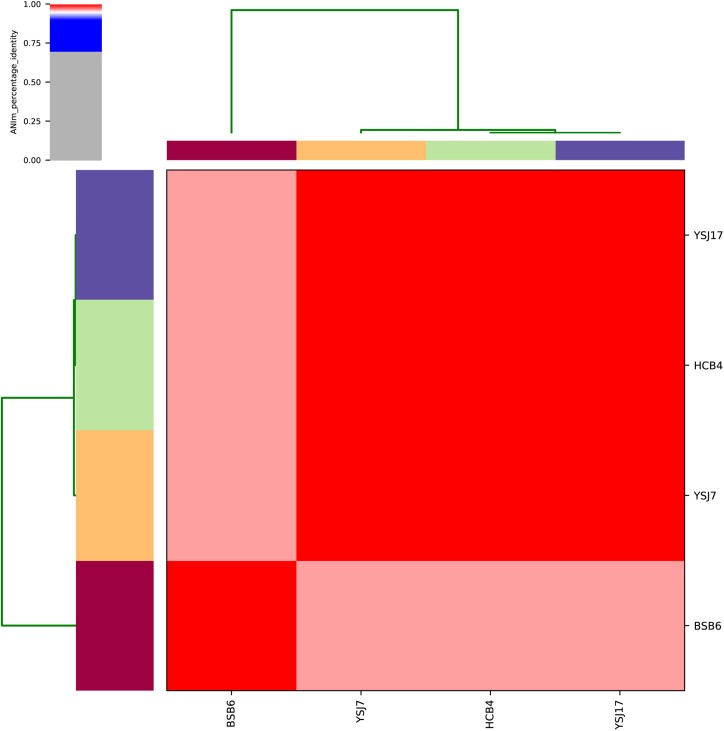
Average nucleotide identity (ANI) analysis of the four *vanG*-carrying *S. suis* isolates using whole-genome sequences.

**FIGURE 2:**
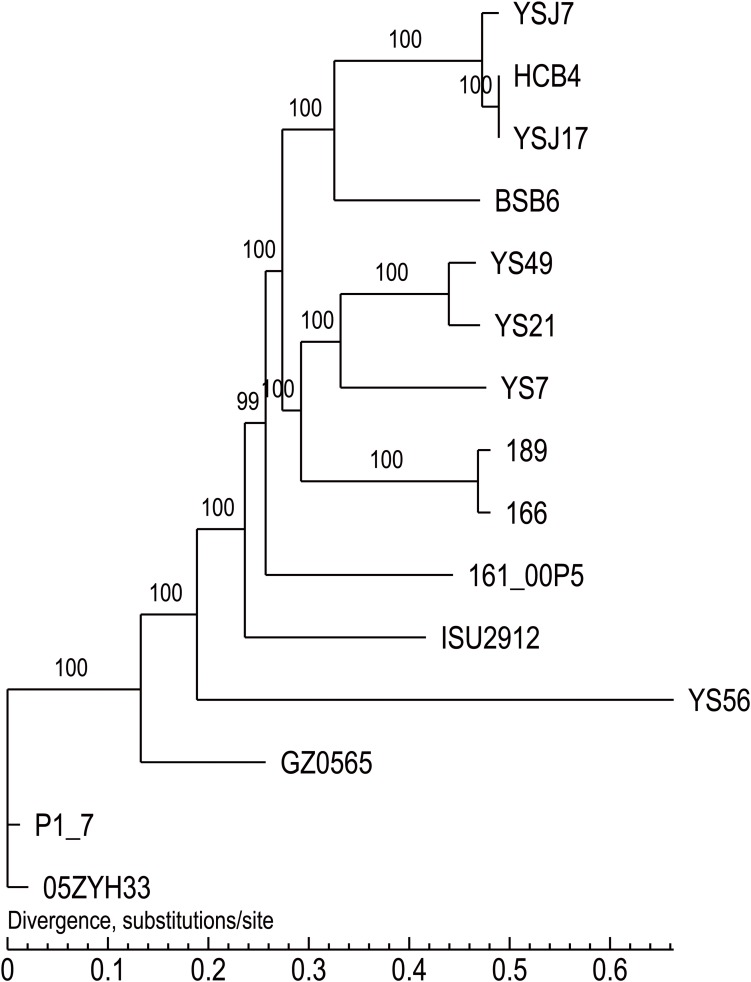
The cSNPs-based phylogenetic trees of the three *vanG*- and *optrA*-carrying *S. suis* isolates generated by comparison with other NCL1 strains from GenBank, and representative genomes of *S. suis* serotype 2 (P1/7 and 05ZYH33), serotype 9 (GZ0565), and serotype 24 (BSB6).

Whole-genome sequencing analysis of the three isolates showed that they were all assigned to multi-locus sequence type ST1071. ANI analysis showed that YSJ17 had 0.9998 and 0.9987 average identity to HCB4 and YSJ7, respectively, but less similar to serotype 24 strain BSB6 (0.9659 of ANI) (Figure 1). Further SNP analysis showed only 1 and 343 cSNPs of HCB4 and YSJ7 to YSJ17, respectively. According to the capsular polysaccharide synthesis locus, the isolates can be classified to a novel capsular polysaccharide loci (NCL) type NCL1 (Okura et al., 2014; Qiu et al., 2016). Remarkable, *S. suis* NCL1 strains have been frequently isolated from both diseased and healthy pigs (Qiu et al., 2016; Zheng et al., 2017). To analyze the evolution of the three *vanG*- and *optrA*-carrying NCL1 isolates, a cSNPs-based phylogenetic tree was generated by comparison with other NCL1 strains from GenBank and representative genomes of *S. suis* serotype 2 (P1/7 and 05ZYH33), serotype 9 (GZ0565), and serotype 24 (BSB6). The phylogenetic tree demonstrated that YSJ17, HCB4, and YSJ7 clustered together with serotype 24 strain BSB6 and more distinct to other NCL1 strains (Figure 2).

### AMR Molecular Mechanisms and Mobilome Analysis

BLAST search for the acquired AMR genes revealed the presence of *optrA*, *vanG*, and also the aminoglycoside-modifying enzyme genes *aadE*, *sat4*, *apt*, *aphA3*, and *aac(6′)–aph(2″)*; the tetracycline resistance gene *tet*(O/W/32/O); the macrolide–lincosamide–streptogramin B resistance gene *erm*(B); and the chloramphenicol resistance gene *cat*pC_194_ (Supplementary Table S2). Mutations involved in penicillin resistance were found in *pbp2x* gene encoding penicillin-binding protein PBP2x (Supplementary Figure S1; Ge et al., 2012). Substitutions in GyrA (Ser81–Lys) and ParC (Ser79–Tyr) were observed, which are known to confer fluoroquinolone resistance (Escudero et al., 2007; Yao et al., 2014). No additional characterized genes or mutations for oxazolidinone resistance were observed. These data were in agreement with the AMR phenotype, with the exception of YSJ7 and HCB4, which were susceptible to vancomycin (Table 2).

To characterize further the mobile genetic elements (MGEs) containing these AMR genes, isolate YSJ17 was completely sequenced, with a small plasmid pYSJ17 of 4,065 bp. The YSJ17 chromosome had a size of 2,551,120 bp and encoded 2,489 putative coding sequences. We mapped the ICEs and prophages by comparing 11 publicly available NCL1 strains and serotype 2, 9, and 24 representative strains (Supplementary Figure S2). An ICE (ICE*Ssu*YSJ17) and four prophages (ΦSsuYSJ17-1 to -4) were identified. The size, insertion location, *att* site, and ARGs of the MGEs are summarized in Supplementary Table S2. ICE*Ssu*YSJ17 contained *aadE*, *sat4*, *apt*, *aphA3*, *tet*(O/W/32/O), *erm*(B), and *vanG* operon, while ΦSsuYSJ17-3 harbored *erm*(B), *aphA3*, *aac(6′)–aph(2″)*, *cat*pC_194_, and *optrA*.

To test the transferability of the *vanG*-carrying ICE*Ssu*YSJ17 and *optrA*-carrying ΦSsuYSJ17-3, mating experiments between *S. suis* YSJ17 and recipient strain *S. suis* P1/7RF were performed. However, we could not obtain transconjugant using florfenicol and vancomycin after more than three independent attempts, with donor and recipient at a ratio of approximately 10^8^ and 10^9^ cfu, respectively.

### Genetic Characterization of the *vanG*-Carrying ICE*Ssu*YSJ17

**FIGURE 3:**
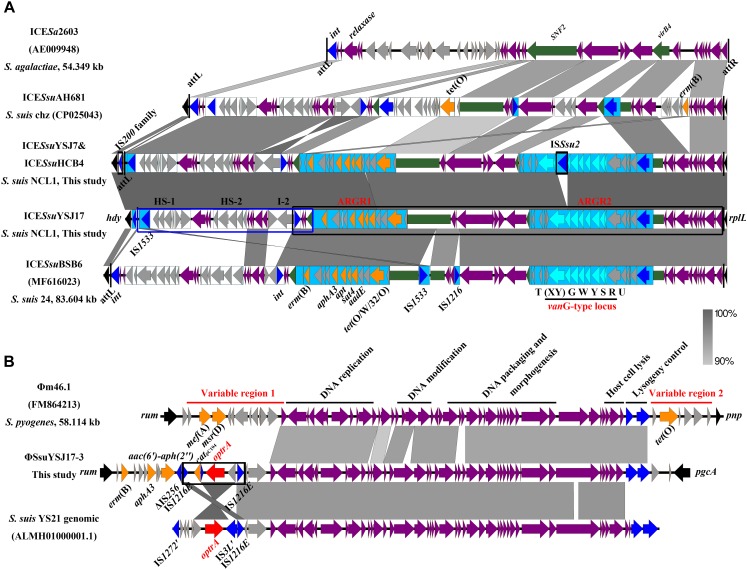
Genetic representation of the *vanG*-carrying ICE*Ssu*YSJ17 and the *optrA*-harboring ΦSsuYSJ17-3. Regions of >90% identity were marked by gray shading. The MGEs’ flanking chromosomal genes were shown in black arrows, integrases/recombinases/transposases were shown in dark blue, core structure genes were in purple arrows, and accessory genes were in light gray arrows. *vanG* operon genes, *optrA*, and other AMR genes were shown in light blue, red, and orange, respectively. **(A)** Comparison of the *S. suis* NCL1 ICE*Ssu*YSJ17 and ICE*Ssu*YSJ7/ICE*Ssu*HCB4 with that of *S. agalactiae* ICE*Sa*2603 (AE009948), *S. suis* Chz ICE*Ssu*AH681 (CP025043), and *S. suis* serotype 24 ICE*Ssu*BSB6 (MF616023). *SNF2* and *vriB4* genes that were inserted with ARGR1 and ARGR2 were highlighted in green. ARGR1, ARGR2, intergenic hotspots HS-1 and HS-2, and insertion site I-2 according to ICE*Ssu*BSB6 were indicated. The two-part segments best matched to ICE*Ssu*AH681 and ICE*Ssu*BSB6 were highlighted in the blue box and black box, respectively. Vertical lines indicate the *att* sequence sites. **(B)** Comparison of the *S. suis*ΦSsuYSJ17-3 with *S. pyogenes* Φm46.1 (FM864213) and *S. suis* YS21 genomic sequence (ALMH01000001.1). Horizontal lines indicate the module structure genes and variable region genes. The composite transposon IS*1216E*-*araC*-*optrA*-*hp*-*cat*pC_194_-IS*1216E* was highlighted in the black box.

The *vanG*-carrying ICE*Ssu*YSJ17 was 79,886 bp in length and encoded 89 predicted ORFs. Sequence analysis of ICE*Ssu*YSJ17 indicated that it is a mosaic ICE similar to ICE*Ssu*BSB6 and ICE*Ssu*HA681 and belonged to the ICE*Sa*2603 family (Figure 3A; Ambroset et al., 2015). The majority (right part, nt 21,302–79,886) of ICE*Ssu*YSJ17 was nearly identical to ICE*Ssu*BSB6 (Huang et al., 2018), with only the presence of two extra IS elements, IS*1533* and IS*1216*, in the latter ICE (Figure 3A). This includes ARGI1 containing *erm*(B), *tet*(O/W/32/O), and *aadE*-*apt*-*sat4*-*aphA3* cluster, and ARGI2 carrying *vanG* operon (Huang et al., 2018). The left part (nt 1–24,281) of ICE*Ssu*YSJ17 matched to ICE*Ssu*HA681, with a difference of only an inverted fragment (nt 17,784–21,301). This includes two variable regions (HS-1 and HS-2) and an insertion I-2 previously identified (Huang et al., 2018).

Since *S. suis* YSJ17 and HCB4 were vancomycin susceptible, we tested if the *vanG* operon was intact to explore the genetic basis for the phenotype. As shown in Figure 3A, an IS element of 1,503 bp, which showed 97% identity to IS*Ssu2*, was inserted at the base of the 365 base of *vanXY* gene in strains YSJ17 and HCB4. This, as a result, abolished the expression of *vanYWG(XY)T* resistance operon, although no effect of the *vanURS* regulatory operon was observed.

### Genetic Characterization of the *optrA*-Carrying ΦSsuYSJ17-3 and Detection of the IS*1216E*-*araC*-*optrA*-*hp*-*cat*pC_194_ Translocatable Unit

The 56,723-bp *optrA*-carrying ΦSsuYSJ17-3 was integrated at the 1328 base of *rum* gene, a well-conserved insertion hotspot for other MGEs (Srinivasan et al., 2014; Huang et al., 2016b). Genetic comparison showed that the ΦSsuYSJ17-3 shared conserved prophage backbone to the *mef*(A)- and *tet*(O)-carrying Φm46.1 (Brenciani et al., 2010), the *cad*A/C-*tet*(W) fragment of *S. suis* ΦSsUD.1 (Palmieri et al., 2010), and the genomic sequence of the *optrA*-carrying *S. suis* NCL1 strains YS21/YS49/YS50 (Figure 3B; Huang et al., 2017). Significantly, ΦSsuYSJ17-3 was present in 10 of 11 current available genomes of NCL1 strains, but absent in other *S. suis* serotype strains (Supplementary Figure S2).

**FIGURE 4:**
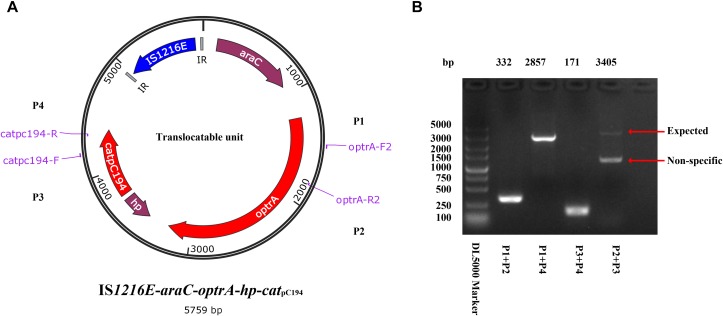
Identification of the translocatable unit of IS*1216E*-*araC*-*optrA*-*hp*-*cat*pC_194_. **(A)** Genetic presentation of the minicircle TU and primers designed. The genes *optrA* and *cat*pC_194_ were shown in red arrows, genes *araC* and hypothetical protein (*hp*) were shown in purple, and *tnp*_*IS*_*_1216E_* was shown in blue. Two IR sequences flanking IS*1216E* were shown in gray. **(B)** PCR products using P1–P4 primer pairs by a TU verification PCR method. The products were further sequenced and confirmed the circular form presented in **(A)**.

Different from the genetic context of *optrA* in plasmids and chromosomes of *E. faecalis*, which usually occupy the *optrA*-*erm*(A)-like or *fexA*–*optrA* resistance cluster (He et al., 2016), and in *S. suis* YS21/YS49/YS50, which occurred in an 8.1-kb *optrA* and *erm*(A)-like containing element or a 7.4-kb *optrA*-carrying fragment (Huang et al., 2017), a 6,568-bp size composite transposon organized in IS*1216E*-*araC*-*optrA*-*hp*-*cat*pC_194_-IS*1216E* structure was present on ΦSsuYSJ17-3. A TU verification PCR using *optrA-F2/catpC194-R* and *optrA-R2/catpC194-F* pairs amplified a product of 2,857 and 3,405 bp, respectively (Figure 4). This confirmed the formation of TU of IS*1216E*-*araC*-*optrA*-*hp*-*cat*pC_194_ with a size of 5,759 bp in all three isolates.

### Inducible Vancomycin Resistance Assays

**FIGURE 5:**
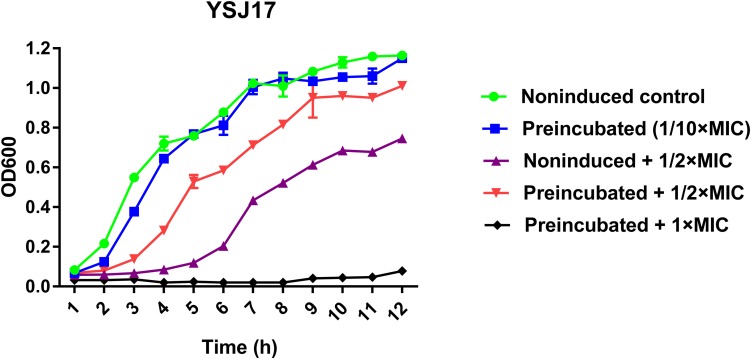
Inducible vancomycin resistance phenotype assay. Strain YSJ17 was tested for inducible vancomycin resistance by preincubation of 1/10 × MIC (0.2 mg/L) vancomycin for 1 h in Todd–Hewitt broth plus 0.2% yeast extract (THY) prior to dilution back to OD_600_ = 0.05 in the same medium containing 1/2 × MIC (1 mg/L) vancomycin. Growth lag was observed by the non-induced + 1/2 × MIC treatment and partial restoration was observed by the preincubated + 1/2 × MIC treatment.

In order to test if *vanG*-type resistance to vancomycin was inducible in YSJ7, we performed vancomycin resistance phenotype and *vanG* transcription assays. As expected, preincubation with 1/10 × MIC vancomycin shortened the growth lag (Figure 5), and the *vanG* transcription was increased 5.6- and 4.3-fold in the presence of 1/10× or 1/2 × MIC vancomycin, respectively. These suggest that the *S. suis* YSJ17 *vanG*-type resistance to vancomycin was inducible and the *vanG* transcription was similar to that of enterococci (Depardieu et al., 2015; Sassi et al., 2018).

### Virulence of the LVR Strain YSJ17

To evaluate the virulence of the LVR strains, we firstly determined in the genomes for the distribution of 24 virulence-related genes responsible for the virulence of *S. suis* serotype 2 (Fittipaldi et al., 2012; Dong et al., 2015, 2017). As shown in Supplementary Table S3, 16 of 24 virulence-related genes were detected in all three isolates. A previous study suggested that *S. suis* serotype 2 strains carrying six genes (*epf*, *sly*, *rgg*, *endoD*, *comR*, and *scnF*) can be predicted as virulent (Dong et al., 2015). In this study, only *rgg* and *endoD* were detected in all three NCL1 isolates (Supplementary Table S3).

**FIGURE 6:**
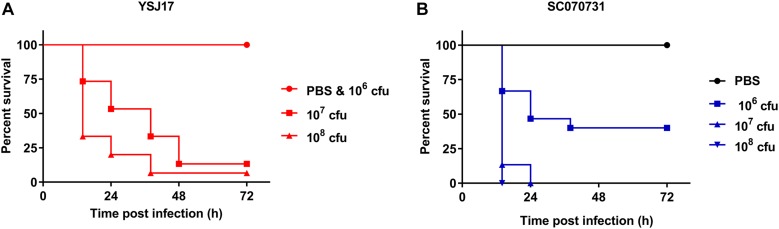
Dose-dependent lethality of zebrafish model infected with **(A)** LVR *S. suis* NCL1 strain YSJ17 and **(B)** the serotype 2 virulent reference strain SC070731. Zebrafish were injected with serial dilutions of 10^5^–10^9^ cfu of *S. suis.* Survival rate was recorded during a 72 h period after infection. Mortality of YSJ17 and SC070731 with a dose of 10^6^–10^8^ cfu was presented.

Although having been frequently isolated from both healthy pigs and lung from diseased pigs, the virulence of NCL1 strains has not been assessed accurately by an animal model. To access this, we measured the virulence of YSJ17 by a zebrafish infection model (Wu et al., 2014). The mortality was 0 and 86.67% after 72-h injection of a dose of 10^6^ and 10^7^ cfu, respectively (Figure 6). While for *S. suis* serotype 2 virulent strain SC070731, the mortality was 40 and 100% with a dose of 10^6^ and 10^7^ cfu, respectively. The LD_50_ was 0.9 × 10^7^ cfu/fish for YSJ17 and 1.2 × 10^6^ cfu/fish for SC070731. Zebrafish infection with the avirulent strain SH040917 showed no mortality. These results suggest that the NCL1 strain YSJ17 was less virulent than *S. suis* serotype 2 virulent strain SC070731. However, the pathogenic mechanism remains to be further explored.

## Discussion

Antimicrobials have been widely used in animals for prevention, treatment, and also as growth promoters. The indiscriminate use of antimicrobials contributes to the emergence of AMR in commensal bacteria, animal pathogens, and also zoonotic pathogens (Mcewen and Fedorka-Cray, 2002). It is of special concern to animal and human health, as resistant bacteria are likely to be transmitted within farm animals and from farm animals to humans through close contact or food chain, as well as AMR genes may be transferred from commensal bacteria to zoonotic pathogens (Mcewen and Fedorka-Cray, 2002; Marshall and Levy, 2011; Thanner et al., 2016). The extensive use of avoparcin in veterinary medicine has led to an increasing incidence of VRE in animals and healthy people during the 1990s (Bager et al., 1997; Klare et al., 1999; Marshall and Levy, 2011). In addition, exclusive use of florfenicol in veterinary medicine could have co-selected the presence of phenicols-oxazolidinones (PhO)-resistant strains (Long et al., 2006; Wang et al., 2015). However, the knowledge addressing the transfer of linezolid- and/or vancomycin-resistant genes from commensal enterococci to zoonotic pathogens is rare. Recently, we characterized the *optrA*-mediated linezolid resistance and *vanG*-type vancomycin resistance in zoonotic pathogen *S. suis* separately (Huang et al., 2017, 2018). The genetic background of *optrA* and *vanG* was highly similar to commensal bacteria enterococci, highlighting the possible transmission from enterococci to *S. suis*. But the prevalence of these genes in *S. suis* has not been investigated.

In the present study, we detected the prevalence of the *vanG* and *optrA* genes in *S. suis* during 2016–2017. The *vanG* gene was detected in *S. suis* NCL1 strains from different cities aside from serotype 24 isolate (Huang et al., 2018), which suggests that *S. suis* may be considered as a possible reservoir for *vanG*, even >20 years after the avoparcin ban in food-producing animals (Bager et al., 1997). In addition, high prevalence of *optrA* was observed in *S. suis*, which may have occurred under florfenicol selection in animal settings (Hao et al., 2016). More alarmingly, to our knowledge, we reported the first LVR isolate carrying the transferrable resistance genes *optrA* and *vanG* operons, which might initially be acquired from enterococci and may contribute to their transfer from *S. suis* to other Gram-positive bacteria (Palmieri et al., 2011; Huang et al., 2016b).

The acquisition and dissemination of AMR genes in streptococci is strongly associated with MGEs, mainly the ICEs and prophages (Palmieri et al., 2011; Huang et al., 2016b). The present study showed the co-location of *vanG* operon with *erm*(B), *tet*(O/W/32/O), and *aadE*-*apt*-*sat4*-*aphA3* cluster on an ICE (ICE*Ssu*YSJ17) of *S. suis* NCL1 strains of different origin (Figure 3A), which is similar to serotype 24 strain of ICESsuBSB6 (Huang et al., 2018). Genetic characterization showed that ICE*Ssu*YSJ17 was a mosaic ICE of the ICE*Sa*2603 family. This family of ICEs are highly prevalent and constitute a diverse group of ICEs associated with AMR in major *Streptococcus* species (Ambroset et al., 2015; Huang et al., 2016b). A variety of AMR determinants for tetracyclines [*tet*(M), *tet*(L), *tet*(O), *tet*(40), and *tet*(O/W/32/O)], macrolides [*erm*(B)], aminoglycosides (*aphA3*, *sat*, *ant6*, and *aadE*), and phenicols (*cat*) have been shown on ICEs of the ICE*Sa*2603 family (Chen et al., 2007; Palmieri et al., 2012; Huang et al., 2016b). Recently, acquisition of the phenicol–oxazolidinone resistance gene *optrA* and the vancomycin resistance gene operon *vanG* within ICE*Sa*2603 family ICE has been observed (Huang et al., 2017, 2018). Particularly, horizontal transfer of ICE*Sa*2603 family ICEs between *Streptococcus* species has been documented (Davies et al., 2009; Haenni et al., 2010; Li et al., 2011; Palmieri et al., 2012). Moreover, tandem recombination of ICE*Ssu*32457 and *S. agalactiae* ICE*Sa*2603 has been reported (Marini et al., 2015). In this study, the transfer of ICE*Ssu*YSJ17 failed. It may be partially due to the inactivation of an essential conjugation protein VirB4 in ICE*Ssu*YSJ17 as in *S. suis* BSB6 (Huang et al., 2018). However, it may also be possible that the experimental conditions were not adapted, with a transfer frequency of <10^–9^ per recipient, as the potential transfer of these genes with the help of other MGEs cannot be ruled out. Thus, the acquisition of *vanG* and other co-located AMR genes within the highly transferable ICE*Sa*2603 family of ICEs may promote the potential transfer of *vanG* operon among Gram-positive cocci.

The present study also showed the co-existence of *optrA* and *cat*pC_194_ as well as *erm*(B), *aphA3*, and *aac(6′)–aph(2″)* on ΦSsuYSJ17-3. Genetic characterization showed that ΦSsuYSJ17-3 was a Φm46.1-like prophage (Brenciani et al., 2010). Φm46.1-like prophage, which was originally reported in *S. pyogenes*, has now been reported in and is transferable to other streptococci (Brenciani et al., 2010; Di Luca et al., 2010; Giovanetti et al., 2014; Huang et al., 2016b). Further analysis showed that the acquisition of *optrA*-*cat*pC_194_ was mediated by an IS*1216* family composite transposon, IS*1216E*-*araC*-*optrA*-*hp*-*cat*pC_194_-IS*1216E* (Figure 3B). IS*1216E* belongs to the IS*6* family and has been increasingly associated with the spread of oxazolidinone resistance genes *cfr*, *optrA*, and *poxtA*; the vancomycin resistance genes *vanA* and *vanM*; the macrolide–lincosamide–streptogramin B resistance genes *erm*(B), *erm*(T), and *lnu*(B); the tetracycline resistance genes *tet*(M), *tet*(L), *tet*(S), and *tet*(S/M); and the aminoglycoside resistance genes *spw* and *aadE* in Gram-positive cocci (Xu et al., 2010; Barile et al., 2012; Liu et al., 2012; Ciric et al., 2014; He et al., 2016; Li et al., 2016; Di Sante et al., 2017). As composite transposons have a potential to excise and form as a TU, we developed a TU verification PCR and detected the mini-circle TU of IS*1216E*-*araC*-*optrA*-*hp*-*cat*pC_194_ (Figure 4), probably *via* IS*1216E*-mediated recombination, highlighting the role of IS*1216E* in the spread of *optrA*-*cat*pC_194_ genes.

## Conclusion

In conclusion, to our knowledge, this is the first report of the co-occurrence of *optrA* and *vanG* operons in Gram-positive bacteria. The acquisition and persistence of *optrA-* and *vanG*-carrying *S. suis* in pigs may contribute to the potential transfer of these resistance genes to other Gram-positive bacteria.

## Data Availability

The *S. suis* YSJ17 complete genome and its plasmid pYSJ17 have been deposited in GenBank (Accession Nos. CP032064 and CP032065), and the *S. suis* YSJ7 and HCB4 draft genomes have been deposited in GenBank (Accession Nos. QXEQ00000000 and QXEP00000000).

## Author Contributions

JH and LW developed the concept and designed the experiments. JH, FD, XL, and DD performed the experiments and collected the data. JH and LW prepared the manuscript. All authors have contributed to, and seen and approved the manuscript.

## Conflict of Interest Statement

The authors declare that the research was conducted in the absence of any commercial or financial relationships that could be construed as a potential conflict of interest.

## Footnotes

1 https://github.com/widdowquinn/pyani
