# Supplementary material for: Characterization of a Linezolid- and Vancomycin-Resistant Streptococcus suis Isolate That Harbors optrA and vanG Operons
Source: Front Microbiol. 2019 Sep 10;10:2026. doi: 10.3389/fmicb.2019.02026 (PMC6746840; doi:10.3389/fmicb.2019.02026)
Supplement: Supplementary file 1 [file Table_1.DOCX]

**Table S1 Primers used in this study.**

| **Primers** | **Sequence (5′ to 3′)** | **Reference** |
| --- | --- | --- |
| vanG-F | GAAGATGGTACTTTGCAGGGCA | (Domingo et al., 2005) |
| vanG-R | AGCCGCTTCTTGTATCCGTTTT | (Domingo et al., 2005) |
| cfr-F | TGAAGTATAAAGCAGGTTGGGAGTCA | (Kehrenberg and Schwarz, 2006) |
| cfr-R | ACCATATAATTGACCACAAGCAGC | (Kehrenberg and Schwarz, 2006) |
| cfrB-F | CCGCATCCGTGAACTAACAG | This study |
| cfrB-F | GCTGGTTGGGAGTCATTTTGT | This study |
| cfrC-F | GGTGAAACTGTTGTGGAGAT | (Tang et al., 2017) |
| cfrC-R | AGTTTCCGTAACTGTCGTTT | (Tang et al., 2017) |
| optrA-F | AGGTGGTCAGCGAACTAA | (Wang et al., 2015) |
| optrA-R | ATCAACTGTTCCCATTCA | (Wang et al., 2015) |
| poxtA-F | TCCACAAAGGATGGGTTATG | (Huang et al., 2019b) |
| poxtA-R | ATGCCCGTATTGGTTATCTC | (Huang et al., 2019b) |
| For *optrA*-*cat*pC194 element circular form (translocatable unit) detection | | |
| optrA-F2 | TTCAAGTAATAAAGACCGGTA | This study |
| optrA-R2 | TATCTTAGTTCGCTGACCAC | This study |
| catpc194-F | TGGACTTCATTTACTGGGTT | This study |
| catpc194-R | GTTCATAAACAATCCTGCAT | This study |
| For ARGI2 element circular form detection | | |
| vanG-INV-1 | TTCTTGCCTTTGTTAGTCGTT | This study |
| vanG-INV-2 | CTCTTGCCATTAGTGTTGCAT | This study |
| For RT-PCR | | |
| vanR_G_-F | AGCAAATGAGGTGGATTTGG | (Huang et al., 2018) |
| vanR_G_-R | CTGCGATTTGACTCTTGCTG | (Huang et al., 2018) |
| vanG-F | TGATGCTGCCATAGAGTTGG | (Huang et al., 2018) |
| vanG-R | CGCTTGACAGTTCGATTTCA | (Huang et al., 2018) |

**Table S2** MGEs identified in *S. suis* YSJ17

|  | Size (bp) | Location | *att* site ^a^ | Putative *att* sequence | ARGs |
| --- | --- | --- | --- | --- | --- |
| **ICESsuYSJ17** | **79,886** | **1,172,462-1,252,349** | ***rplL* (3')** | **GTCACTCTTAAATAA** | ***aadE*, *sat4*,*apt*, *aphA3*, *tet*(O/W/32/O), *erm*(B), *vanG*** |
| ΦSsuYSJ17-1 | 43,842 | 778,631-822,472 | *tRNA-Arg* (3') | ATTCCTGCAGGGGAGATG |  |
| ΦSsuYSJ17-2 | 42,266 | 1,060,259-1,102,524 | *SSU0916* (3') | GTGTTATAATAGAATAGT |  |
| **ΦSsuYSJ17-3** | **56,723** | **1,442,158-1,498,880** | ***rum* (3')** | **GA** | ***erm*(B), *aphA3*, *aac(6')-aph(2'')*, *cat*_pC194_, *optrA*** |
| ΦSsuYSJ17-4 | 37,290 | 1,834,545-1,871,834 | *tRNA-Thr* (3') | ATGCTAGTTGCC |  |
| pYSJ17 | 4,065 | Plasmid |  |  |  |

^a^ Corresponding to *S. suis* P1/7.

**Table S3** Presence of 24 virulence-associated genes for *S. suis* serotype 2 in *optrA*- and *vanG*-positive *S. suis* NCL1 isolates

| **Strain** ^a^ | **P1/7** | **SC070731** | **05HAS68** | **YSJ17** | **YSJ7** | **HCB4** |
| --- | --- | --- | --- | --- | --- | --- |
| Serotype | 2 | 2 | 2 | NCL1 | NCL1 | NCL1 |
| Predicted virulence ^a^ | virulent | virulent | avirulent | moderate | moderate | moderate |
| **Virulence related genes** ^b^ | **locus_tag** | **% Identity to genes in P1/7** | | | | |
| **>1.epf ^c^** | **SSU0171** | **99** |  |  |  |  |
| **>2.sly** | **SSU1231** | **100** |  |  |  |  |
| >3.mrp | SSU0706 | 99 | 99 |  |  |  |
| >4.fbps | SSU1311 | 100 | 96 | 94 | 94 | 94 |
| **>5.rgg** | **SSU1789** | **100** |  | **93** | **93** | **93** |
| >6.ofs | SSU1474 | 100 | 84 |  |  |  |
| >7.srtA | SSU0925 | 100 | 99 | 97 | 97 | 97 |
| >8.pgdA | SSU1448 | 100 | 98 | 98 | 98 | 98 |
| >9.gdh | SSU0234 | 100 | 97 | 97 | 97 | 97 |
| >10.gapdh | SSU0153 | 100 | 99 | 99 | 99 | 99 |
| >11.iga | SSU0879 | 100 | 93 | 91 | 91 | 91 |
| **>12.endoD** | **SSU1715** | **99** |  | **98** | **98** | **98** |
| >13.ciaRH | SSU0944 | 100 | 97 | 91 | 91 | 91 |
| >14.salKR | N | N |  |  |  |  |
| >15.manN | SSU1585 | 100 | 100 | 87 | 87 | 87 |
| >16.purD | SSU0032 | 99 | 94 | 94 | 94 | 94 |
| >17.dppIV | SSU0187 | 100 | 94 | 92 | 92 | 92 |
| **>18.comR** | **SSU0049** | **100** | **99** |  |  |  |
| **>19.scnF** | **SSU0835** | **100** |  |  |  |  |
| >20.sspA | SSU0757 | 100 | 98 | 98 | 98 | 98 |
| >21.neuB | SSU0535 | 100 | 100 |  |  |  |
| >22.dltA | SSU0596 | 100 | 99 | 98 | 98 | 98 |
| >23.stp | SSU0383 | 100 | 100 | 98 | 98 | 98 |
| >24.lysM | SSU0215 | 99 | 94 | 98 | 98 | 98 |

^a^ P1/7 and SC070731 were isolated from pig with meningitis and were virulent in zebrafish infection model. 05HAS68 was isolated from the tonsil of a healthy pig and was avirulent in pathogen-free (SPF) piglets. The three *optrA*- and *vanG-*carrying NCL1 isolates exhibited moderate virulence in zebrafish infection model.

^b^ Virulence related genes for *S. suis* serotype 2 were according to previous study.^7-9^

^c^ *S. suis* serotype 2 strains carrying these six virulence genes (underlined) could be predicted as virulent as reported previously.^7^

**
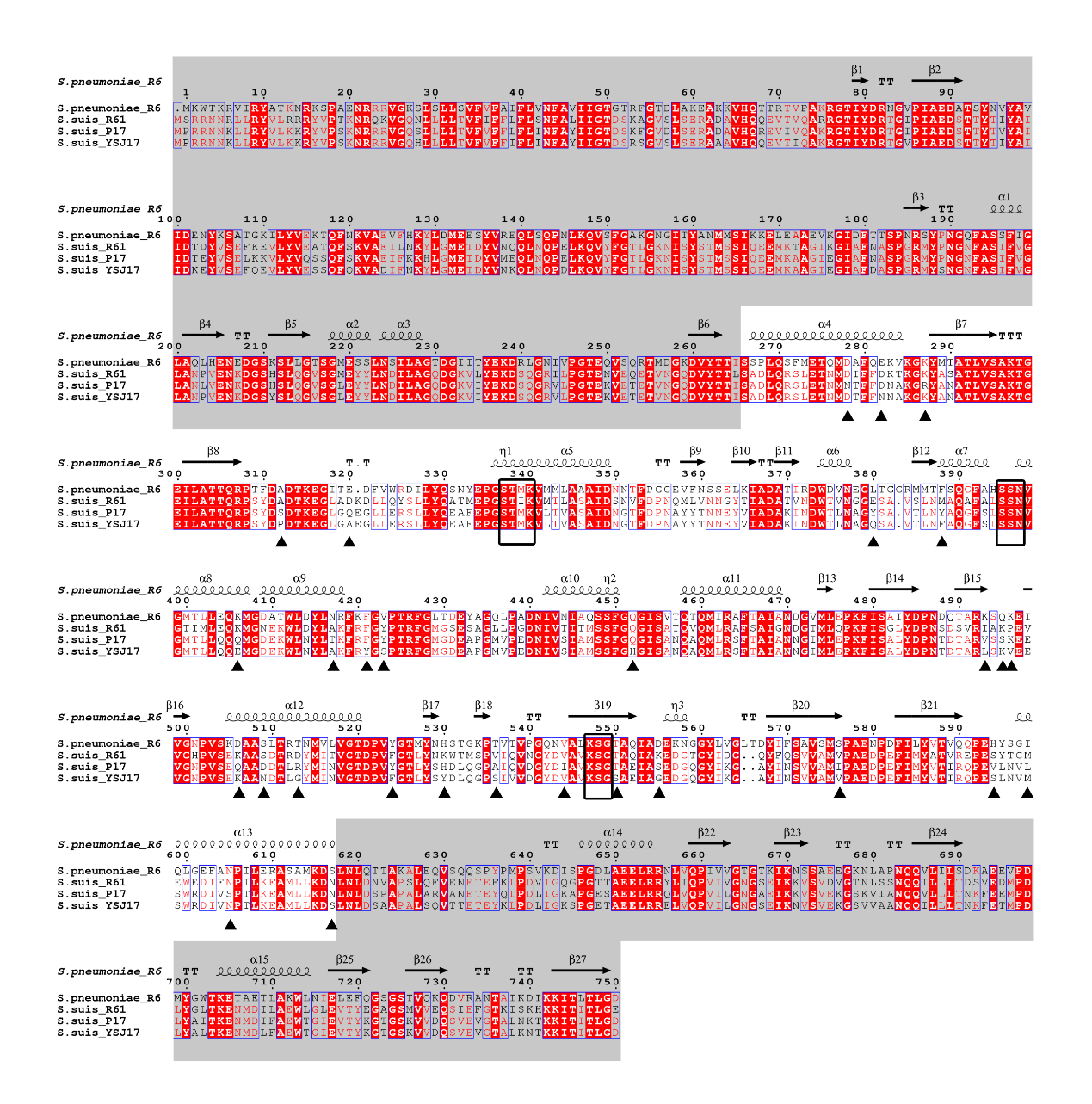
Figure S1** Alignments of the PBP2X of *S. suis* YSJ17 (penicillin-resistant and cefuroxime-sensitive) with *S. suis* R61 (penicillin- and cefuroxime-resistant), P1/7 (penicillin- and cefuroxime-sensitive) and *S. pneumoniae* R6. Secondary structure of *S. pneumoniae* R6 were shown on top of the sequences. Regions other than the transpeptidase domain were marked by grey shading. Amino acid difference in transpeptidase domain between strains YSJ17 and P1/7 were marked by black triangle under sequences.

**
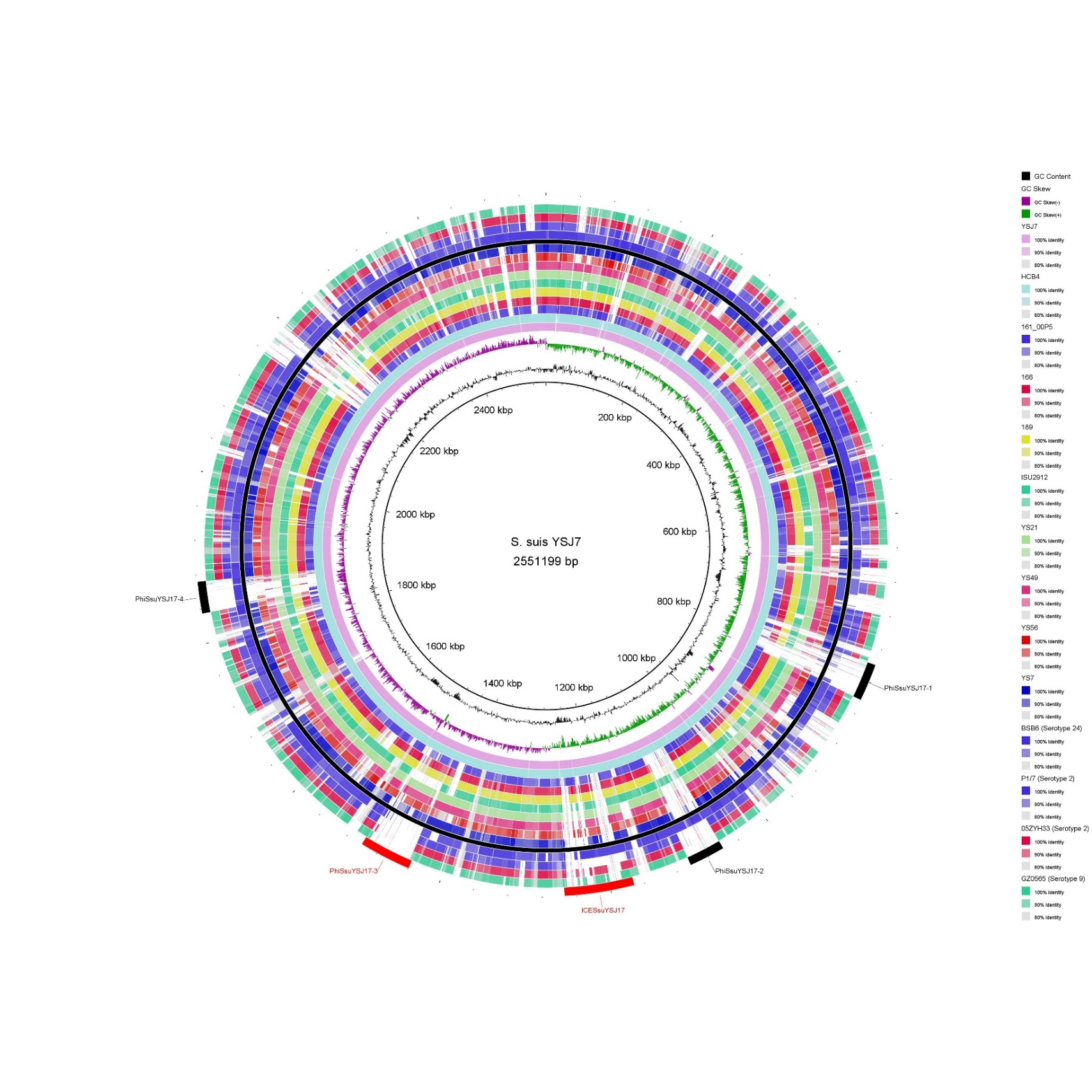
**

**Figure S2** Circular visualization of the comparison of *S. suis* NCL1 strain YSJ17 with 14 representative strains. These included NCL1 strains YSJ7 and HCB4 in this study, 8 public available NCL1 strains from Genbank, and the complete genome of *S. suis* serotype 24 strain BSB6, serotype 2 strains P1/7 and 05ZYH33, and serotype 9 strain GZ0565. The identified ICE and prophages were shown on outward.

**References**

1. Domingo MC, Huletsky A, Giroux R et al. High prevalence of glycopeptide resistance genes *vanB*, *vanD*, and *vanG* not associated with enterococci in human fecal flora. *Antimicrob Agents Chemother* 2005; **49**: 4784-6.

2. Kehrenberg C, Schwarz S. Distribution of florfenicol resistance genes *fexA* and *cfr* among chloramphenicol-resistant *Staphylococcus* isolates. *Antimicrob Agents Chemother* 2006; **50**: 1156-63.

3. Tang Y, Dai L, Sahin O et al. Emergence of a plasmid-borne multidrug resistance gene *cfr*(C) in foodborne pathogen *Campylobacter*. *J Antimicrob Chemother* 2017; **72**: 1581-8.

4. Wang Y, Lv Y, Cai J et al. A novel gene, *optrA*, that confers transferable resistance to oxazolidinones and phenicols and its presence in *Enterococcus faecalis* and *Enterococcus faecium* of human and animal origin. *J Antimicrob Chemother* 2015; **70**: 2182-90.

5. Huang J, Wang M, Gao Y et al. Emergence of plasmid-mediated oxazolidinone resistance gene *poxtA* from CC17 Enterococcus faecium of pig origin. *J Antimicrob Chemother* 2019; doi.org/10.1093/jac/dkz250.

6. Huang J, Chen L, Li D et al. Emergence of a *vanG*-carrying and multidrug resistant ICE in zoonotic pathogen *Streptococccus suis*. *Vet Microbiol* 2018; **222**: 109-13.

7. Dong W, Ma J, Zhu Y et al. Virulence genotyping and population analysis of *Streptococcus suis* serotype 2 isolates from China. *Infect Genet Evol* 2015; **36**: 483-9.

8. Dong W, Zhu Y, Ma Y et al. Multilocus sequence typing and virulence genotyping of *Streptococcus suis* serotype 9 isolates revealed high genetic and virulence diversity. *FEMS Microbiol Lett* 2017; **364**.

9. Fittipaldi N, Segura M, Grenier D et al. Virulence factors involved in the pathogenesis of the infection caused by the swine pathogen and zoonotic agent *Streptococcus suis*. *Future Microbiol* 2012; **7**: 259-79.
